# The Emerging Pathogen Candida auris: Growth Phenotype, Virulence Factors, Activity of Antifungals, and Effect of SCY-078, a Novel Glucan Synthesis Inhibitor, on Growth Morphology and Biofilm Formation

**DOI:** 10.1128/AAC.02396-16

**Published:** 2017-04-24

**Authors:** Emily Larkin, Christopher Hager, Jyotsna Chandra, Pranab K. Mukherjee, Mauricio Retuerto, Iman Salem, Lisa Long, Nancy Isham, Laura Kovanda, Katyna Borroto-Esoda, Steve Wring, David Angulo, Mahmoud Ghannoum

**Affiliations:** a Center for Medical Mycology, Case Western Reserve University, and University Hospitals Cleveland Medical Center, Cleveland, Ohio, USA; b Astellas Pharma Global Development, Inc., Northbrook, Illinois, USA; c Scynexis Inc., Jersey City, New Jersey, USA

**Keywords:** Candida auris, SCY-078, virulence, biofilm

## Abstract

Candida auris, a new multidrug-resistant Candida spp. which is associated with invasive infection and high rates of mortality, has recently emerged. Here, we determined the virulence factors (germination, adherence, biofilm formation, phospholipase and proteinase production) of 16 C. auris isolates and their susceptibilities to 11 drugs belonging to different antifungal classes, including a novel orally bioavailable 1,3-β-d-glucan synthesis inhibitor (SCY-078). We also examined the effect of SCY-078 on the growth, ultrastructure, and biofilm-forming abilities of C. auris. Our data showed that while the tested strains did not germinate, they did produce phospholipase and proteinase in a strain-dependent manner and had a significantly reduced ability to adhere and form biofilms compared to that of Candida albicans (*P* = 0.01). C. auris isolates demonstrated reduced susceptibility to fluconazole and amphotericin B, while, in general, they were susceptible to the remaining drugs tested. SCY-078 had an MIC_90_ of 1 mg/liter against C. auris and caused complete inhibition of the growth of C. auris and C. albicans. Scanning electron microscopy analysis showed that SCY-078 interrupted C. auris cell division, with the organism forming abnormal fused fungal cells. Additionally, SCY-078 possessed potent antibiofilm activity, wherein treated biofilms demonstrated significantly reduced metabolic activity and a significantly reduced thickness compared to the untreated control (*P* < 0.05 for both comparisons). Our study shows that C. auris expresses several virulence determinants (albeit to a lesser extent than C. albicans) and is resistant to fluconazole and amphotericin B. SCY-078, the new orally bioavailable antifungal, had potent antifungal/antibiofilm activity against C. auris, indicating that further evaluation of this antifungal is warranted.

## INTRODUCTION

The Centers for Disease Control and Prevention recently published an alert that emerging multidrug-resistant Candida auris is causing invasive infections (1). Originally isolated in 2008 from a Japanese patient's ear canal (2), C. auris has been reported to cause serious invasive infections (e.g., candidemia) with a high associated rate of mortality (approaching approximately 60%) (3). C. auris has caused serious infections globally, including in Japan, South Korea, India, Kuwait, South Africa, Pakistan, and the United Kingdom and, more recently, in Venezuela, Colombia, and the United States (2, 4–12). Of these C. auris infections, the majority have been secondary nosocomial infections (4, 9, 12, 13). A high percentage of clinical C. auris strains demonstrate resistance to fluconazole and show variable resistance to other antifungals belonging to the three major classes of clinically available antifungals (azoles, polyenes, echinocandins), thereby limiting treatment options (1, 3–6, 9–16).

To gain insight into this emerging Candida species, investigators conducted studies to characterize its virulence factors (e.g., phospholipase, proteinase, ability to form biofilms) (8, 17). Although these studies are informative, they examined only a limited number of C. auris strains. Here we studied 16 different C. auris isolates obtained from patients in Japan, India, South Korea, and Germany and (i) characterized their morphology and virulence factors (those that have been described for Candida species, particularly Candida albicans, e.g., germination, adherence, biofilm formation, and phospholipase and proteinase production), (ii) determined their susceptibilities to 11 antifungals belonging to different antifungal classes, including a novel orally bioavailable 1,3-β-d-glucan synthesis inhibitor (SCY-078) with demonstrated activity against multidrug-resistant Candida spp., and (iii) evaluated the effect of SCY-078 on the growth, ultrastructure, and biofilm-forming ability of C. auris (18).

## RESULTS

### C. auris does not form chlamydospores.

C. auris failed to form chlamydospores after growth on cornmeal agar for 3 days at 30°C. In contrast, C. albicans ATCC 14053 (the positive control) produced chlamydospores on cornmeal agar (data not shown).

### Virulence factors.

**FIG 1:**
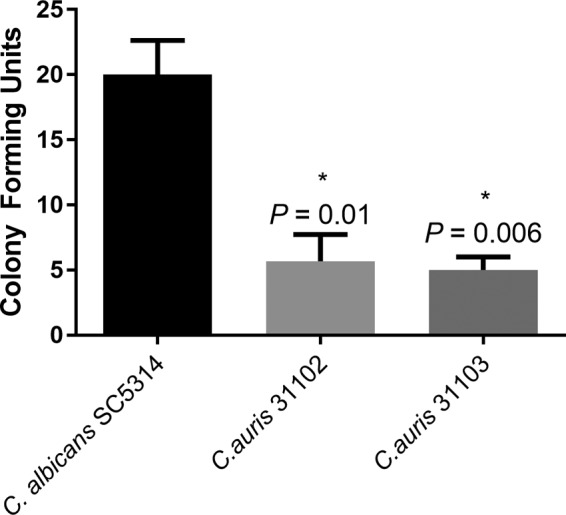
Comparison of adherence of C. auris strains. The ability of Candida species to adhere to a silicon elastomer catheter as a representative substrate was assessed. Cells were allowed to adhere to silicone elastomer discs, washed, and overlaid with Sabouraud dextrose agar, and the number of CFU adhering to the substrate was counted after incubation at 37°C for 18 to 24 h. The number of adherent C. auris cells was significantly less than that for C. albicans (positive control) (*P* ≤ 0.01). *, *P* value compared to the value for C. albicans.

Our data showed that the C. auris strains tested did not germinate when incubated with fetal bovine serum. In contrast, C. albicans ATCC 14053 germinated profusely (>90% within 2 h). Moreover, the evaluation of adherence using two representative C. auris isolates (MRL 31102 and MRL 31103) revealed that the C. auris strains exhibited a significantly reduced ability to adhere to catheter material compared to C. albicans (*P* < 0.05) (Fig. 1).

The tested C. auris strains produced phospholipase and proteinase in a strain-dependent manner. As shown in Table 1, 37.5% of the tested C. auris strains (6/16 isolates) possessed phospholipase activity and 64% (9/14 isolates) tested positive for secreted proteinase activity (19). The level of proteinase production by the 9 strains ranged from 1.2 to 5.3 ng/ml (Table 1).

**TABLE 1:** Phospholipase and proteinase activities of C. auris isolates

Strain	Species	Phospholipase activity		Proteinase activity (ng/ml)
		Class	*P_z_* value^*a*^	
SC5314	C. albicans	Control (++)	0.66	
MRL 31102	C. auris	−	1.00	
MRL 31103	C. auris	−	1.00	
CBS 10913	C. auris	−	1.00	0.0
CBS 12372	C. auris	+	0.90	0.0
CBS 12373	C. auris	−	1.00	1.4
CBS 12766	C. auris	+	0.90	0.0
CBS 12767	C. auris	−	1.00	0.0
CBS 12768	C. auris	+	0.90	2.3
CBS 12770	C. auris	++	0.78	1.8
CBS 12771	C. auris	−	1.00	4.7
CBS 12772	C. auris	−	1.00	0.0
CBS 12773	C. auris	+	0.91	1.2
CBS 12774	C. auris	−	1.00	2.8
CBS 12775	C. auris	+	0.91	1.6
CBS 12776	C. auris	−	1.00	5.3
CBS 12777	C. auris	−	1.00	3.2

a ++, *P_z_* = <0.89 (strong phospholipase activity); +, *P_z_* = 0.90 to 0.99 (weak phospholipase activity); −, *P_z_* = 1 (no phospholipase activity).

**FIG 2:**
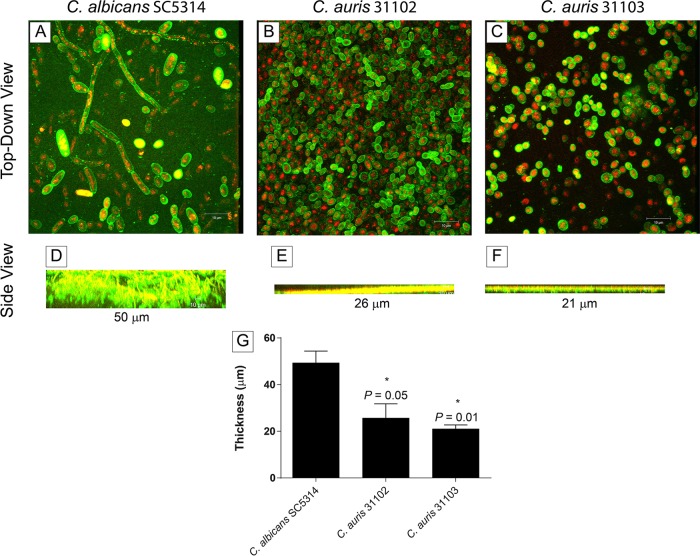
Formation of biofilms by C. albicans and C. auris strains. Confocal scanning laser micrographs show top-down three-dimensional views (A to C) and side views (D to F) of biofilms formed by C. albicans (A, D), C. auris MRL 31102 (B, E), and C. auris MRL 31103 (C, F). Magnifications, ×100. (G) Thickness of biofilms formed by the tested isolates. *, *P* value compared to the thickness of C. albicans biofilms. A *P* value of <0.05 was considered significant. All experiments were done in triplicate, and data represent means ± SDs. C. albicans SC5314 showed a highly heterogeneous architecture of biofilms with yeast cells and hyphae embedded within the extracellular matrix, while C. auris biofilms had minimal extracellular matrix and were significantly thinner than C. albicans biofilms.

**FIG 3:**
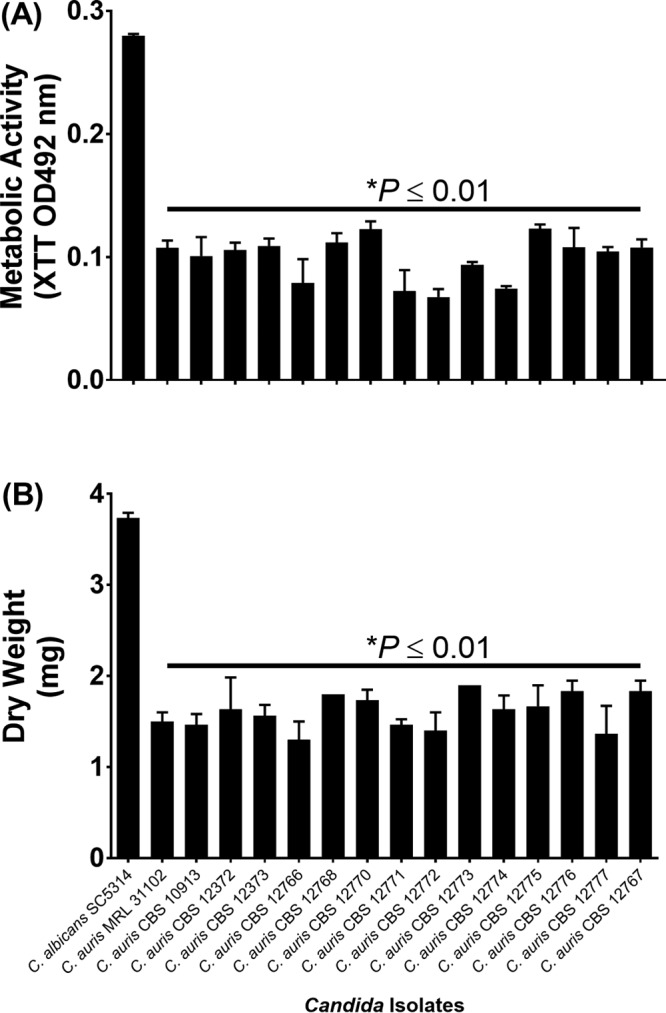
Quantification of biofilms formed by C. albicans and C. auris strains. The metabolic activity (A) and dry weight (B) of the biofilms formed by C. albicans, C. auris MRL 31102 (control), and 14 CBS C. auris strains are shown. *, *P* value compared to the results for C. albicans. A *P* value of <0.05 was considered significant. All experiments were done in triplicate, and the data in both plots represent means ± SDs. C. auris biofilms had significantly reduced metabolic activity and biomass compared to those of C. albicans biofilms.

Analysis of biofilm formation by C. auris MRL 31102 and MRL 31103 demonstrated that the biofilms were mainly composed of yeast cells adhering to catheter material (Fig. 2B and C). In contrast, C. albicans SC5314 showed a highly heterogeneous architecture of biofilms with yeast cells and hyphae embedded within the extracellular matrix (Fig. 2A). Moreover, C. auris biofilms, unlike C. albicans biofilms, had a limited amount of extracellular matrix. Furthermore, the thickness of C. auris biofilms was significantly less than that of C. albicans biofilms (range, 21 to 26 μm for C. auris biofilms versus 50 μm for C. albicans biofilms; *P* ≤ 0.05) (Fig. 2D to G). Biofilm quantitation on the basis of metabolic activity and biomass revealed that the C. auris isolates tested (*n* = 15) formed significantly less biofilm than C. albicans SC5314 (Fig. 3A and B) (*P* < 0.05 for both comparisons).

### C. auris growth is similar to C. albicans growth.

**FIG 4:**
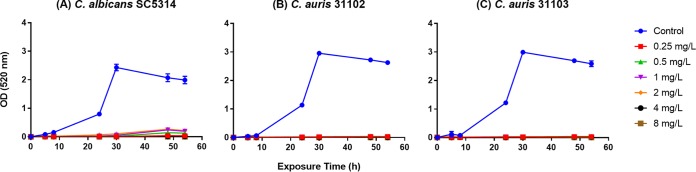
Effect of different concentrations of the antifungal SCY-078 on the growth of C. albicans and C. auris isolates. C. albicans SC5314 (A), C. auris MRL 31102 (B), and C. auris MRL 31103 (C) cells were grown in the presence of the indicated concentrations of SCY-078. At different time points, aliquots were withdrawn and their ODs were determined spectrophotometrically. All experiments were done in triplicate, and the data in all three panels represent means ± SDs. SCY-078 inhibited the growth of the C. albicans and C. auris strains.

Growth curve analysis of the control C. auris and C. albicans isolates showed that the two Candida species had similar growth patterns, reaching stationary phase within approximately 20 h (Fig. 4). The doubling times for C. albicans SC5314, C. auris MRL 31102, and C. auris MRL 31103 were 101.22 ± 5.26, 105.62 ± 12.83, and 107.00 ± 7.14 min, respectively (mean ± standard deviation [SD]; *P* > 0.05 for all comparisons).

### Antifungal susceptibility profile of C. auris.

As shown in Table 2, the MIC_90_ of SCY-078 was 1 mg/liter, which was similar to the MIC_90_ of caspofungin (CAS) and micafungin (MFG) (1 mg/liter for both) and within 2 dilutions of the MIC_90_ of anidulafungin (AFG; 0.25 mg/liter). The MIC_90_ of SCY-078 was also similar to that of flucytosine (5FC; MIC_90_ = 1 mg/liter) and lower than that of amphotericin B (AMB; MIC_90_ = 4 mg/liter). Antifungal susceptibility testing of azoles showed that isavuconazole (ISA) was the most active agent tested (MIC_90_ = 0.125 mg/liter), followed by posaconazole (POS; MIC_90_ = 0.5 mg/liter), itraconazole (ITC; MIC_90_ = 1 mg/liter), and voriconazole (VRC; MIC_90_ = 2 mg/liter), while fluconazole (FLC) was the least active azole (MIC_90_ > 64 mg/liter) against the tested isolates (Table 3).

**TABLE 2:** *In vitro* susceptibilities of C. auris isolates to 1,3-β-d-glucan synthesis inhibitors

Strain	MIC^*a*^ (mg/liter)			
	SCY-078	AFG	CAS	MFG
MRL 31102	0.5	0.25	0.5	0.25
MRL 31103	0.5	0.25	0.5	0.25
CBS 10913	1	0.125	0.5	2
CBS 12372	1	0.125	0.5	1
CBS 12373	1	0.125	0.5	1
CBS 12766	0.5	0.125	0.5	1
CBS 12767	1	0.125	0.5	1
CBS 12768	1	0.125	0.5	2
CBS 12770	1	0.125	0.5	1
CBS 12771	1	0.25	0.5	1
CBS 12772	1	0.25	1	1
CBS 12773	1	0.25	1	1
CBS 12774	2	0.125	0.25	1
CBS 12775	1	0.125	0.25	1
CBS 12776	1	0.125	1	1
CBS 12777	1	0.125	1	1
Range	0.5–2	0.125–0.25	0.25–1	0.25–2
MIC_50_	1	0.125	0.5	1
MIC_90_	1	0.25	1	1

a MICs were determined at 24 h.

**TABLE 3:** *In vitro* susceptibilities of C. auris isolates to azoles, 5FC, and AMB

Strain	MIC*^a^* (mg/liter)									
	5FC (48 h)	AMB		FLC		ISA (24 h)	ITC (48 h)	POS (48 h)	VRC	
		24 h	48 h	24 h	48 h				24 h	48 h
MRL 31102	0.5	4	4	>64	>64	0.031	0.5	0.5	0.125	0.5
MRL 31103	0.5	4	4	>64	>64	0.031	0.5	0.25	0.125	0.5
CBS 10913	1	1	2	1	2	0.004	<0.063	0.25	<0.063	<0.063
CBS 12372	0.5	1	2	2	>64	0.25	1	1	0.5	2
CBS 12373	0.5	1	2	16	>64	0.125	1	0.25	0.25	2
CBS 12766	0.5	4	4	32	>64	0.125	1	0.5	0.5	0.5
CBS 12767	1	2	4	2	>64	0.016	0.5	0.25	0.5	0.5
CBS 12768	0.5	4	4	32	>64	0.125	1	0.25	0.5	0.5
CBS 12770	0.5	4	4	32	>64	0.25	0.5	0.5	0.5	2
CBS 12771	0.5	4	4	8	>64	0.063	0.5	0.5	1	1
CBS 12772	1	8	8	>64	>64	0.125	0.5	0.5	0.5	1
CBS 12773	0.5	2	8	>64	>64	0.063	1	1	0.5	0.5
CBS 12774	0.5	2	4	>64	>64	0.063	0.5	0.5	1	2
CBS 12775	0.5	2	4	1	>64	0.016	0.5	0.25	0.5	2
CBS 12776	0.5	2	4	1	>64	0.063	0.5	0.25	0.5	0.5
CBS 12777	0.5	0.5	4	1	>64	0.063	0.5	0.25	1	1
Range	0.5 to 1	0.5 to 8	2 to 4	1 to >64	2 to >64	0.004 to 0.25	<0.063 to 1	0.25 to 1	<0.063 to 1	<0.063 to 2
MIC_50_	0.5	2	4	16	>64	0.063	0.5	0.25	0.5	0.5
MIC_90_	1	4	4	>64	>64	0.125	1	0.5	1	2

### SCY-078 inhibits the growth of C. auris and C. albicans.

Having shown that C. auris had low MIC values of SCY-078, we examined the ability of this drug to inhibit the growth of this yeast. Exposure of C. auris and C. albicans to SCY-078 (at concentrations ranging from 0.25 to 8 mg/liter) led to the complete inhibition of growth of this pathogenic fungus (Fig. 4).

### SCY-078 disrupted the ultrastructure of C. auris.

**FIG 5:**
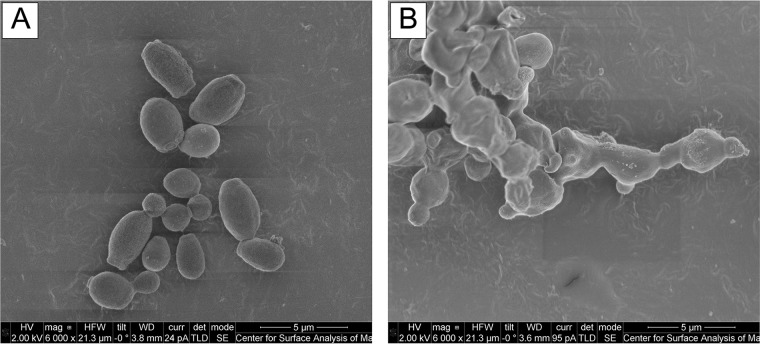
Scanning electron micrograph of C. auris treated with no drug (control) (A) and with SCY-078 at 1× MIC (0.5 mg/liter) (B). Cells were exposed to no drug (control) or SCY-078 at 1× MIC overnight at 35°C and then fixed in 2% glutaraldehyde and processed for scanning electron microscopy. Untreated control C. auris cells had a well-defined, oval-shaped yeast morphology as well as several budding yeasts (A). In contrast, cells exposed to SCY-078 (at a concentration of 1× MIC) exhibited a severely distorted yeast cell topography with cells fused together, indicating that the cells were unable to divide (B). Magnifications, ×6,000.

We used scanning electron microscopy to determine the effect of SCY-078 on the ultrastructure of C. auris. As can be seen in Fig. 5A, untreated control C. auris cells had a well-defined, oval-shaped yeast morphology as well as several budding cells. In contrast, cells exposed to SCY-078 (at a concentration of 1× MIC [0.5 mg/liter]) exhibited a severely distorted yeast cell topography with cells fused together, indicating that the cells were unable to divide (Fig. 5B).

### SCY-078 possesses activity against C. auris biofilms.

**FIG 6:**
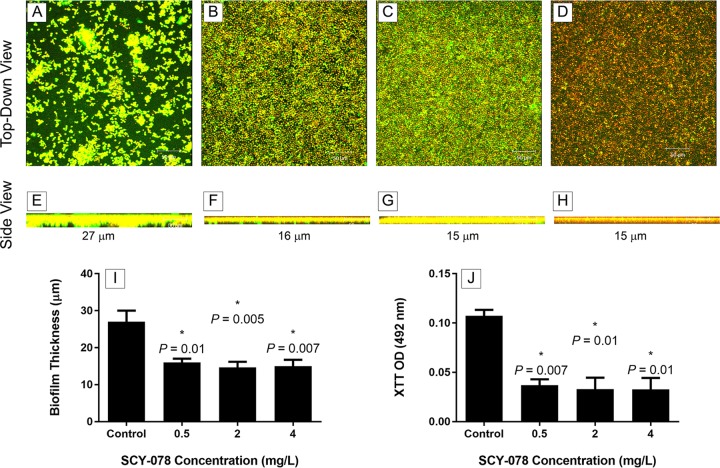
Confocal scanning laser microscopy analyses of the effect of SCY-078 on biofilms formed by C. auris. Biofilms formed by C. auris MRL 31102 were exposed to no drug (control) (A, E) or SCY-078 at different concentrations: 0.5 mg/liter (B, F), 2 mg/liter (C, G), or 4 mg/liter (D, H). Top-down views (A to D) and side views (E to H) of untreated and treated biofilms are shown. Magnifications, ×25. (I and J) The thickness (I) and metabolic activity (J) of untreated (control) and SCY-078-treated biofilms. *, *P* value compared to the results for the untreated control (no drug). A *P* value of <0.05 was considered significant. All experiments were done in triplicate, and the bars represent means ± SDs. SCY-078 exhibited potent activity against biofilms formed by C. auris strains.

Resistance to antifungals is a hallmark phenotype of biofilms. To determine whether SCY-078 possesses antibiofilm activity, we exposed mature-phase C. auris biofilms to a range of drug concentrations (0.5, 2, and 4 mg/liter). Metabolic activity and confocal scanning laser microscopy data showed that SCY-078 reduced C. auris biofilms significantly at all concentrations tested (*P* < 0.05) (Fig. 6) (19). Unlike untreated cells, which showed an intense green fluorescence (resulting from concanavalin A [ConA] binding to polysaccharides), C. auris biofilms treated with SCY-078 showed yeast cells with reduced fluorescence, particularly at an SCY-078 concentration of 4 mg/liter (Fig. 6D and H). Additionally, at all tested drug concentrations, the metabolic activity and thickness of the biofilms were reduced significantly compared to those for untreated control biofilms (*P* < 0.05) (Fig. 6I and J).

## DISCUSSION

In the current study, we examined 16 different C. auris isolates obtained from different parts of the world; characterized the major Candida virulence factors (including germination, adherence, biofilm formation, and phospholipase and proteinase production); determined their susceptibilities to 11 drugs belonging to different antifungal classes, including a novel orally bioavailable 1,3-β-d-glucan synthesis inhibitor (SCY-078); and evaluated the effect of SCY-078 on the growth, morphology, and ultrastructure of C. auris and its ability to form biofilms.

Germination, adherence, phospholipase and proteinase production, and biofilm formation are major virulence factors known to contribute to Candida pathogenesis and have been well characterized in C. albicans by a number of investigators, including our group (20–24). Few studies have investigated these virulence factors in C. auris, and those that have been conducted have been limited to strains from a single geographical areas or have focused on one strain (7, 17). Our data showed that C. auris, unlike C. albicans, is devoid of the ability to germinate, form hyphae, or produce chlamydospores. Our data confirm and extend the observations by others since we analyzed isolates from different geographic regions (3, 4, 17). Additionally, we showed for the first time that C. auris exhibits a minimal ability to adhere to silicone elastomer relative to C. albicans. Since the ability of Candida to adhere to catheter surfaces is important in causing catheter-associated candidiasis, the weak adherence ability of C. auris suggests that it likely plays some role in catheter-associated candidiasis but not a large one, in contrast to C. albicans and Candida parapsilosis, which are known to cause such infections (25).

Our findings demonstrated that C. auris phospholipase production was strain dependent and that phospholipase production was detected in 37.5% of the isolates tested. In general, the tested C. auris strains that produced this enzyme tended to have weak phospholipase activity. Only one C. auris strain (CBS 12770) had phospholipase activity comparable to that of C. albicans, a known high phospholipase producer (26). Kumar et al. (17) recently reported the isolation of an azole-resistant C. auris strain from a vulvovaginitis patient and showed that this strain exhibited strong phospholipase activity (ratio of the colony diameter to the colony diameter plus the precipitation zone [*P_z_*] = 0.72), proteinase activity, and hemolysin activity. Ours is the first study to demonstrate that the production of phospholipase by C. auris is strain dependent, with the majority of isolates being non-phospholipase producers. In general, the number of C. auris strains tested that produced secreted proteinase was higher than the number that produced phospholipase (64% versus 37.5%, respectively). However, similar to the ability to produce phospholipase, the ability of C. auris to produce secreted proteinase was strain dependent. Interestingly, we observed that strains that were high proteinase producers were not necessarily high phospholipase producers and vice versa (Table 1).

Our results showed that the tested C. auris strains had the ability to form biofilms. However, these biofilms were significantly thinner than the biofilms formed by C. albicans (the C. auris biofilms were approximately 50% the thickness of the C. albicans biofilms). A variation in the ability of different C. auris strains to form biofilms was noted, though these differences were not statistically significant. Our results are in disagreement with those of Oh et al. (7), who tested 15 C. auris isolates and reported that they did not produce biofilms. The differences between our data and those of Oh et al. (7) could be attributed to the use of different media and the fact that they did not use silicon elastomer as a substrate, nor did the investigators treat the plastic surfaces with fetal bovine serum (27). Alternatively, all the isolates used in the study of Oh et al. (7) were obtained from patients' ears, while the strains used in this study were mostly from patients with disseminated candidiasis. In addition, Oh et al. (7) used a semiquantitative subjective scale (range, negative result to a score of +5) to report biofilm formation, while we used three separate quantitative measures (metabolic activity, dry weight, and biofilm thickness).

In general, the ability of C. auris to express the various virulence factors is much weaker than that of C. albicans, suggesting that this emerging species is not as virulent as the latter species. Borman et al. (8) attempted to address the virulence of C. auris by using a Galleria mellonella larva model. Their data showed that C. auris had the same pathogenicity as C. albicans and C. tropicalis and was more virulent (*P* < 0.05) than other Candida spp. (e.g., C. glabrata, C. parapsilosis, C. krusei). However, unpublished data from our center confirm the poor ability of C. auris to infect and disseminate in mice compared to C. albicans (data not shown). We showed that it is critical to immunocompromise the mice and use a large inoculum (3 × 10^7^ yeast cells/animal) to successfully infect the mice (unpublished data). Therefore, our data suggest that the multidrug-resistant phenotype of C. auris comes with a major fitness cost.

The clinical significance of C. auris appears to reside in its ability to develop resistance to multiple commonly used antifungal agents, leading to infections with high rates of mortality (2–6, 8, 9, 13–16). Therefore, the identification of agents that are effective against this species is critical. Our data demonstrated that C. auris responded differently to various antifungals. Among the azoles, all but one isolate showed reduced susceptibility to fluconazole (MIC, >64 mg/liter), with the other azoles showing variable antifungal activity and isavuconazole being the most active. The only isolate tested in this study to have a low fluconazole MIC was CBS 10913, which, interestingly, was the only strain isolated from a nonblood source (2). Additionally, caspofungin, micafungin, and anidulafungin showed similar activities against the tested isolates. These findings are in agreement with those of recent studies from other investigators, who also reported that C. auris isolates were generally resistant or less susceptible to azoles but susceptible to echinocandins (2–6, 8, 9, 13, 14, 16). Our finding that amphotericin B demonstrated less activity against C. auris strains also agrees with those of others reporting high MICs for this agent against C. auris (9, 16, 28). Our data show that the C. auris isolates tested in our study exhibited multidrug resistance against fluconazole and amphotericin B. Moreover, some isolates also exhibited high MIC values for voriconazole and itraconazole.

To identify new antifungals that may be affective against C. auris, in this study we tested SCY-078, a new 1,3-β-d-glucan synthesis inhibitor, which has been shown to possess potent activity against various Candida spp. An added advantage of SCY-078 is that it is the first orally bioavailable 1,3-β-d-glucan synthesis inhibitor (29). Our data showed that SCY-078 has potent antifungal activity against the C. auris isolates tested. Further, SCY-078 showed growth inhibition and antibiofilm activity against this emerging species. Moreover, C. auris cells exposed to SCY-078 exhibited a severely distorted yeast cell topography and failed to divide. Other investigators showed that increasing concentrations of caspofungin treatment altered the morphology of various Candida species (30, 31). These authors showed that caspofungin treatment of cells affected the morphology of Candida, resulting in cells with an increased size, a lack of distinct rings around the budding site, and the absence of filamentation in C. albicans. Unlike caspofungin, treatment with SCY-078 led to the inhibition of cell division, suggesting that, in addition to the inhibition of 1,3-β-d-glucan synthesis, this drug may have a separate target or may affect this enzyme through yet undefined unique interactions.

In summary, our study showed that C. auris expresses several virulence factors, albeit to a lesser extent than C. albicans and in a strain-dependent manner. We demonstrated that SCY-078 is a potent drug and could be an important addition to the antifungal armamentarium to treat patients with infections caused by this multiresistant species.

## MATERIALS AND METHODS

### Isolates tested.

The following C. auris strains were used in this study: 2 C. auris isolates (MRL 31102 and MRL 31103) obtained from a 68-year-old male enrolled in a recent candidemia trial at a German site and 14 C. auris isolates from the CBS-KNAW Fungal Biodiversity Centre, Utrecht, the Netherlands (CBS 10913, CBS 12372, CBS 12373, CBS 12766 to CBS 12768, and CBS 12770 to CBS 12777) that were isolated from patients living in Japan, India, and South Korea (Table 4). To identify the MRL 31102 and MRL 31103 isolates, we originally utilized the yeast identification tool API 20C AUX. However, we were unable to identify the strains with a high percentage (>85%) of certainty. Moreover, since C. auris is also often misidentified as Candida haemulonii, Candida famata, Candida sake, or Rhodotorula glutinis using typical identification methods, including the API 20C AUX tool and the Vitek 2 system, we used DNA typing, employing the internal transcribed spacer 1 (ITS1) and ITS4 regions of the fungal ribosome (2–6, 12–15). Identification was performed using a BLAST algorithm search against the sequences in the UNITE database (32). This analysis allowed us to identify MRL 31102 and MRL 31103, which had 89 and 99% homology with the C. auris strains in the UNITE database, respectively, as C. auris. In the current study, two strains of C. albicans (SC5314 and ATCC 14053), obtained from the American Type Culture Collection (Manassas, VA), were used as comparators. C. albicans was selected as a comparator because its virulence factors have been well characterized and it is responsible for nearly 50% of all Candida infections.

**TABLE 4:** C. auris isolates used in this study

Strain	Species	Isolation source	Country of isolation
MRL 31102	C. auris	Blood	Germany
MRL 31103	C. auris	Blood	Germany
CBS 10913	C. auris	Ear	Japan
CBS 12372	C. auris	Blood	South Korea
CBS 12373	C. auris	Blood	South Korea
CBS 12766	C. auris	Blood	India
CBS 12767	C. auris	Blood	India
CBS 12768	C. auris	Blood	India
CBS 12770	C. auris	Blood	India
CBS 12771	C. auris	Blood	India
CBS 12772	C. auris	Blood	India
CBS 12773	C. auris	Blood	India
CBS 12774	C. auris	Blood	India
CBS 12775	C. auris	Blood	India
CBS 12776	C. auris	Blood	India
CBS 12777	C. auris	Blood	India

### Ability of C. auris to form chlamydospores.

Since chlamydospores are utilized to help distinguish fungal types (33), we evaluated the ability of the C. auris isolates to exhibit this morphotype. Briefly, an isolated colony of C. auris was inoculated onto a cornmeal agar plate (Becton Dickinson, Sparks, MD, USA) to make a lawn. A coverslip was applied to the inoculated area in order to create microaerophilic conditions, and the plate was incubated at 30°C for 3 days with protection from light. The plate was subsequently examined for chlamydospore formation under white light at a ×20 magnification. C. albicans ATCC 14053 was used as a positive control.

### Characterization of C. auris virulence factors.

The ability of Candida species to cause infection is attributed to the possession of virulence factors, including the ability to germinate, adhere to host cells, secrete extracellular enzymes such as phospholipase and proteinase, and form biofilms (18, 34, 35). To date, the characterization of C. auris virulence factors is limited.

### (i) Ability to germinate.

The ability of C. auris to form germ tubes was assessed using the germ tube test (36–38). Briefly, an isolated colony of C. auris, taken from a 24-h culture grown on potato-dextrose agar (Becton Dickinson, Sparks, MD, USA), was inoculated into 0.5 ml of fetal bovine serum (Fisher Thermo Scientific, Cleveland, OH, USA) and incubated for 2 h at 37°C. Next, 10 μl of fetal bovine serum was then transferred onto a glass slide, a coverslip was added, and the slide was examined using phase-contrast microscopy at a ×20 magnification.

### (ii) Adherence assay.

The ability of Candida species to adhere to indwelling medical devices and host tissues is a known virulence factor. Since silicon elastomer is used extensively in central venous catheters, in this study, we evaluated the ability of C. auris isolates to adhere to silicon elastomer, which was used as a representative catheter material.

To determine the ability of C. auris to adhere to silicon elastomer surfaces, yeast cells were inoculated into 10 ml yeast nitrogen base broth (Becton Dickinson, Sparks, MD, USA) and incubated overnight at 37°C. The cells were then washed twice with Hanks' buffered salt solution, adjusted to 5 × 10^3^ cells/ml, and further diluted to obtain a cell suspension of 250 cells/ml using the same buffer. Silicon elastomer discs (diameter, 1.2 cm) were transferred to 12-well tissue culture plates, to which 2 ml of cell suspension (prepared as described above) was added. Next, the plates were incubated at 37°C for 30 min to allow the cells to adhere to the discs. After incubation, discs containing adherent cells were transferred to a fresh plate containing 2 ml Hanks' buffered salt solution. Subsequently, the discs were washed twice with Hanks' buffered salt solution and, following aspiration of the buffer, were overlaid with 3 ml of Sabouraud dextrose agar (Becton Dickinson, Sparks, MD, USA). The plates were incubated at 37°C for 18 to 24 h, and the number of C. auris cells adhering to the catheter material was assessed by counting the number of CFU.

### (iii) Phospholipase assay.

The ability of the C. auris strains to secrete phospholipase was determined using a phospholipase activity assay that employed Sabouraud dextrose agar as a medium supplemented with 58.4 g/liter NaCl, 5.5 g/liter CaCl_2_, and 10% egg yolk emulsion (30% stock; Hi-Media, India) as previously described (26, 39). Medium without egg yolk was sterilized at 121°C for 15 min and cooled to 50°C. Egg yolk emulsion was then added, and the agar was dispensed into petri dishes (20 ml/plate). A 10-μl aliquot containing 1 × 10^7^ yeast cells/ml was added to the center of the plates, and the plates were incubated at 37°C for 5 days. Following incubation, the production of phospholipase (indicated by the appearance of a whitish zone of precipitation around the yeast colony) was assessed by measuring the ratio of the colony diameter to the colony diameter plus the precipitation zone (the *P_z_* value). *P_z_* values of ≤0.89, 0.90 to 0.99, and 1 indicate strong, weak, and no phospholipase activity, respectively (26).

### (iv) Proteinase assay.

Proteinase activity assays were performed using a protease fluorescent detection kit (catalog number PF0100; Sigma-Aldrich, St. Louis, MO, USA) according to the manufacturer's specifications (40). The C. auris strains were cultured in 20 ml yeast nitrogen base for 48 h. Following incubation, the supernatant containing secreted proteinases was concentrated, and the volumes were adjusted to the proportion of the total number of CFU. Subsequently, 10 μl of the normalized protein-rich supernatants was tested for proteinase activity using the kit described above.

### (v) Biofilm formation.

The ability of Candida to form biofilms has been linked to catheter-associated infections. Therefore, we evaluated whether C. auris can produce biofilms using our *in vitro* biofilm model as described previously (19). In the current study, we used silicone elastomer as a substrate for biofilm formation since catheters are commonly constructed using this material and silicone elastomer has been used as a substrate in numerous studies investigating catheter-associated biofilms performed by our group and others (41–51). Briefly, silicone elastomer discs (diameter, 1.2 cm) were placed in 12-well tissue culture plates and incubated in fetal bovine serum at 37°C on a rocker for 24 h. The discs were then removed from the fetal bovine serum, immersed in a 4-ml cell suspension with a concentration of 1 × 10^7^ cells/ml, and incubated for 90 min at 37°C (adhesion phase). Subsequently, the discs were transferred to 4 ml yeast nitrogen base medium and incubated for 24 h at 37°C to form mature biofilms (mature phase) (19).

The quantification of the biofilms formed by different strains was performed using a colorimetric metabolic assay (to measure mitochondrial dehydrogenase activity) and dry weight analysis (to measure total biofilm mass, which includes fungal cells and matrix). For evaluation of metabolic activity, discs with biofilms were transferred to 12-well tissue culture plates containing 2,3-bis-(2-methoxy-4-nitro-5-sulfophenyl)-2*H*-tetrazolium-5-carboxanilide (XTT; 12.5 μg/ml) and menadione (1 μM) in phosphate-buffered saline and incubated at 37°C for 3 h as described previously (19). Next, the biofilms were scraped, transferred into a tube, and centrifuged at 3,000 rpm for 15 min. The absorbance of the resulting supernatant was measured spectrophotometrically at 492 nm. To determine dry weight (biomass), the pellet obtained from the centrifugation step described above was resuspended in phosphate-buffered saline and filtered through a preweighed filter (pore size, 0.2 μm; Millipore, Billerica, MA, USA), dried at 37°C for 48 h, and weighed (19). The biomass of the biofilms was calculated from the difference in the weight of the preweighed filters.

To examine the morphology and thickness of the formed biofilms, confocal scanning laser microscopy was used. Briefly, following biofilm formation, the silicon elastomer discs on which the biofilms were developed were transferred to a 35-mm-diameter glass-bottom petri dish (MatTek Corp., Ashland, MA, USA) and incubated for 45 min at 37°C in 2 ml of phosphate-buffered saline containing the fluorescent stains FUN-1 (10 μM) and concanavalin A (ConA; 25 mg/liter)-Alexa Fluor 488 conjugate (both dyes from Molecular Probes, Inc., Eugene, OR). FUN-1 (excitation wavelength, 543 nm; emission wavelength, 560 nm) is converted to orange-red cylindrical intravacuolar structures by metabolically active cells, and ConA (excitation wavelength, 488 nm; emission wavelength, 505 nm) fluoresces green when bound to glucose and mannose residues of fungal polysaccharides (present in the cell wall and biofilm matrix). After incubation with the dyes, the silicon elastomer discs were flipped and the biofilms were examined using a Zeiss LSM510 confocal scanning laser microscope (Carl Zeiss, Inc.). To determine the structure of the biofilms, a series of horizontal (*x-y*) optical sections with a thickness of 0.9 μm were taken at 0.44-μm intervals throughout the full length of the biofilm. Confocal images of green (ConA) and red (FUN-1) fluorescence were simultaneously collected using a multitrack mode (19).

### Determination of susceptibility profiles of C. auris strains.

To establish the susceptibility profiles of the C. auris strains, broth microdilution susceptibility testing was performed in accordance with the guidelines in the Clinical and Laboratory Standards Institute (CLSI) M27-A3 document (52). RPMI 1640 medium with 3-(*N*-morpholino)propanesulfonic acid (RPMI), an inoculum of 0.5 × 10^3^ to 2.5 × 10^3^ cells/ml, and incubation at 35°C were used. The activities of 11 antifungals against the C. auris isolates were tested, including SCY-078 (Scynexis, Jersey City, NJ, USA), a novel orally bioavailable 1,3-β-d-glucan synthesis inhibitor, and currently available antifungals amphotericin B and anidulafungin (Pfizer Pharmaceuticals, New York, NY, USA), caspofungin (Merck Co., Kenilworth, NJ, USA), fluconazole, flucytosine, and isavuconazole (Astellas Pharma US, Inc., Northbrook, IL, USA), itraconazole and micafungin (Astellas Pharma, Inc., Tokyo, Japan), posaconazole, and voriconazole (AMB, FLC, 5FC, ITC, POS, and VRC were obtained from Sigma-Aldrich, St. Louis, MO, USA). MIC panels were incubated for 24 h (SCY-078, AMB, AFG, CAS, FLC, ISA, MFG, and VRC) or 48 h (5FC, AMB, FLC, ITC, POS, and VRC). MIC endpoints were determined visually as the lowest concentration of drug that resulted in the complete inhibition of growth (AMB) or a decrease of growth by ≥50% relative to that of the growth control (SCY-078, AFG, CAS, FLC, 5FC, ISA, ITC, MFG, POS, VRC) (52). In all instances, MIC plates were prepared using reagent-grade powders, as directed by CLSI (52).

### Effect of SCY-078 on the growth of C. auris and C. albicans.

Evaluation of the ability of a drug to inhibit microbial growth is conventionally determined either by counting the number of CFU, which is an indicator of cell viability, or by turbidity measurement (using a spectrophotometer), which indicates cell density but does not differentiate between live and dead cells (52, 53). In this study, we used the spectrophotometric approach to evaluate the ability of SCY-078 to inhibit Candida growth as described previously (54). Briefly, cells were harvested from 18- to 24-h-old cultures, washed twice with phosphate-buffered saline, and adjusted to 5 × 10^5^ cells/ml in 50-ml conical tubes containing RPMI alone (with no drug as a growth control) or 0.5×, 1×, 2×, 4×, 8×, or 16× MIC of SCY-078. Medium alone with no drug or yeast cells was used as a blank. All tubes were incubated at 37°C. At different time points (0, 5, 8, 24, 30, 48, and 54 h), aliquots were taken and their optical densities (ODs) were determined spectrophotometrically at 520 nm. A growth curve showing cell inhibition temporally was constructed.

### Scanning electron microscopy.

The effect of SCY-078 on C. auris morphology and ultrastructure was determined using scanning electron microscopy as described previously (19). Briefly, the C. auris strains were exposed overnight to 1× MIC (0.5 mg/liter) of SCY-078 at 35°C. Next, 200 μl of a cell suspension was fixed in 2% glutaraldehyde, and the fixed cell suspension was incubated at 4°C for 48 h. After fixation, the samples were processed and dried. The processed samples were coated with palladium for 60 s and viewed with a Nova NanoLab 200 FEG-SEM/FIB scanning electron microscope in high-vacuum mode at 2.00 kV.

### Effect of SCY-078 on C. auris biofilms.

To evaluate the activity of SCY-078 against C. auris biofilms, discs with mature biofilms were transferred to wells containing different concentrations of SCY-078 (range, 0.5 to 4 mg/liter). Following 48 h of incubation, the metabolic activities of the biofilms were measured using the XTT reduction assay as described above. Images and the thicknesses of biofilms growing in the presence or absence of drug were captured using confocal scanning laser microscopy, also as described above (19).

### Statistical analysis.

Statistical analyses of all the data were performed using GraphPad Prism (version 6) software. The treated groups were compared to the untreated control groups using unpaired *t* tests, and a *P* value of <0.05 was considered statistically significant. All experiments were done in triplicate. Doubling times were calculated using R, the statistical programming language (https://cran.r-project.org/).

### Accession number(s).

The sequences of C. auris strains MRL 31102 and MRL 31103 have been deposited in GenBank under accession numbers KY514262 and KY514058, respectively.
